# Numerical Solutions for a Model of Tissue Invasion and Migration of Tumour Cells

**DOI:** 10.1155/2011/452320

**Published:** 2010-12-30

**Authors:** M. Kolev, B. Zubik-Kowal

**Affiliations:** ^1^Department of Mathematics and Computer Science, University of Warmia and Mazury, Zolnierska 14, 10-561, Olsztyn, Poland; ^2^Department of Mathematics, Boise State University, 1910 University Drive, Boise, ID 83725, USA

## Abstract

The goal of this paper is to construct a new algorithm for the numerical simulations of the evolution of tumour invasion and metastasis. By means of mathematical model equations and their numerical solutions we investigate how cancer cells can produce and secrete matrix degradative enzymes, degrade extracellular matrix, and invade due to diffusion and haptotactic migration. For the numerical simulations of the interactions between the tumour cells and the surrounding tissue, we apply numerical approximations, which are spectrally accurate and based on small amounts of grid-points. Our numerical experiments illustrate the metastatic ability of tumour cells.

## 1. Introduction

The analysis of data obtained from the World Health Organization (WHO) [1] and the UN [2] databases shows that, at present, cancer is and probably will remain to be among the leading causes of death worldwide [3–5] being surpassed only by cardiovascular diseases. According to the data provided by the WHO, cancer disease is the cause of the death of roughly six million people yearly [1]. This explains the major significance of the fight against the malignant conditions, which includes prevention [6], cure [7, 8], and cancer research.

Tumour development is a very complex multistep process involving many intracellular and extracellular phenomena which are strongly nonlinear and time varying [4, 9–11]. Genomic changes as well as microenvironmental factors such as the * extracellular matrix* (ECM), various growth factors, and substrate concentrations have been shown to play a major role in the process of carcinogenesis [12].

Generally, tumours can be classified as *benign* and * malignant*. The growth of benign tumours is self-limiting and their cells tend to stay in the same place. Malignant tumours may grow without limitations and their constituent cells are prone to migrate or metastasize to other parts of the organism [13–15]. The ability of malignant cancer to invade the local tissue and to spread throughout the organism is their most insidious and dangerous property. Metastasis is the predominant cause of most cancer deaths [14, 16, 17].

The process of metastasis includes angiogenesis and invasion. Tumour angiogenesis (rapid growth of blood vessels near the tumour cells) is induced by a secretion of various growth factors such as vascular endothelial growth factor (VEGF). These vessels facilitate the influx of oxygen and other nutrients needed for the development of the cancer [18]. The process of angiogenesis is followed by invasion and penetration of cancer cells into surrounding tissues and possibly by dissemination of cancer cells through blood vessels. Thus, tumour cells can be carried to a distant site of the body. There they can implant and initiate the development of a secondary tumour [14, 16, 19]. An important role in the process of cancer invasion is performed by * matrix degradative enzymes* (MDEs) such as metallo-proteases (MMPs). They are produced by tumour cells and digest the ECM, which enables the migration of cancer cells through the tissue [13, 14, 17].

In the last half century, many mathematical models describing the process of tumourigenesis have been the subject of active research. Mathematical and computational methods have contributed to clarifying the factors that are sufficient to explain experimental and clinical data, to defining these factors in precise terms and to suggesting experiments for calculation of these factors [20]. In addition, analyses and simulations of mathematical models have been used for the reduction of the amounts of costly experiments needed for the development of therapies [21, 22]. It is strongly believed that mathematical and computational methods will play a significant role in cancer research in the future. They may improve the understanding of some complicated features and details of tumour evolution as well as be effectively used in clinical laboratories, by means of appropriate model-based decision support systems [4]. We refer the readers to special issues [23–27] for more complete bibliography regarding the applications of mathematical and computational methods to cancer research.

Gatenby and Gawlinski present one of the first models of tumour invasion in the papers [28, 29]. Gatenby [28] considers the competition between healthy host cells and modified (tumour) cells and proposes and analyses several models formulated in terms of ordinary differential equations. Gatenby and Gawlinski [29] present a reaction-diffusion model for the investigation of the role of the alteration of the microenvironmental acidity induced by cancer cells for their invasion into the organism. Subsequently, the series of papers, among others [30–43], have appeared offering models and detailed analysis of diverse features of cancer invasion. In this paper, we study the continuum models of avascular tumour growth investigated by Chaplain et al. (cf., e.g., [31, 34–37]). The first model of this series is proposed in Anderson et al. [31]. The authors consider three major variables involved in the process of cancer invasion, namely, cancer cells, ECM, and MDEs. In order to study in detail mainly the influence of the surrounding tissue on the process of migration of tumour cells, the proliferation of the latter is not included in the continuum model. The authors analyse numerically in one and two dimensions the impact of ECM gradients resulting from the destruction of ECM by MDE and the role of haptotaxis on cancer invasion. An extension of this model is presented in Chaplain and Anderson [34] who consider the role of oxygen as a nutrient for the tumour cells. The authors propose also a new model equation for endogenous inhibitors, such as tissue inhibiting MMPs, that can neutralize MDEs. We include this equation in our model (8), see Section 2 below. The model of Chaplain and Anderson [34] has been further developed by Lolas [37] and Chaplain and Lolas [35, 36] who have considered terms describing chemotaxis, proliferation of cancer cells and reestablishment of the ECM. Lolas [37] examines a variety of continuum models, in particular incorporating the effects of just chemotaxis, and haptotaxis, and their combination, and so forth. One of the conclusions of the author is that the mechanism of chemotaxis without haptotaxis cannot lead to a successful cancer invasion if there is no proliferation of tumour cells and reestablishment of ECM. Further novel ordinary differential equations that describe the cancer cell proliferation and the remodeling of the extracellular matrix re-establishment function allowing the incorporation of the plasminogen activation cycle are included in the model of Chaplain and Lolas [36] that also investigates the role of the uPA system for the cancer invasion. uPA inhibitors and plasmin have also been investigated in the model by Chaplain and Lolas [35]. Clear and detailed description of the biological processes observed during the cancer invasion and metastasis is provided in [31, 34–37]. In particular, in these paper, the key stages of the metastatic cascade, the structure and functions of the major constituents of the ECM and the basic representatives of the MDEs participating in the interactions between the healthy and cancer cells are systematically presented on the basis of broad theoretical and experimental bibliography.

In our paper, we propose a different numerical approach than the approach used, for example, in [31, 34–37]. The goal of the paper is to obtain numerical results which are based on small amounts of spatial grid points applied to the model equations so that low-dimensional vectors of data are used to make the numerical computations fast. We construct a new algorithm for the systems [31, 34, 36, 37] by using spectrally accurate approximations to the terms that model the tumour cell random motility, the haptotaxis, the MDE diffusion, and the diffusion of the endogenous inhibitors. Since the algorithm computes the solutions with spectral accuracy, it is based on smaller amounts of spatial grid points than the amounts of grid points used for the less accurate finite difference approximations (strategy applied, e.g., in [31, 34–37]), which consequently saves computational time. The idea of using small amounts of spatial grid point and saving time for computing one solution for one set of parameters, which has to be repeated many times for many sets, is important, for example, for the numerical experiments carrying the goal of estimating parameter values from laboratory data. This idea is applied in [44] to estimate parameter values of one of the models presented in [36, 37] from the * in vivo* experimental data [45] developed by using transgenic mouse models. The numerical approach from [44] is based on a different approximation to the haptotactic term than the approximations used in this paper and our numerical schemes are constructed for systems which are various variants and generalizations of the model investigated in [44]. Furthermore, because of considering different variants of boundary conditions the schemes in this paper differ from that of the paper [44].

Additionally to the model presented in [36, 37] and applied in [44], in this paper, we investigate other models, which are presented in [34] or are combinations of the model equations from [34, 36, 37]. Moreover, in [44], the parameter values are evaluated quantitatively from the laboratory data [45] so that the solutions of the model equations correlate with the data. Contrarily to [44], in this paper, we choose the parameter values qualitatively in order to observe and compare solutions computed with different parameters. This comparison allows to analyse the influence of the parameters on the shape of the solutions and we conclude that complicated interactions between tumour cells, ECM, MDEs, and endogenous inhibitors can be directed by choosing the parameter values. Our sequence of numerical simulations is initiated from the solutions obtained with the parameter values chosen in [34] (for comparison) and next we gradually change the values and analyse their influence on the solutions. Animated graphical visualization of the solutions and how they change according to the parameters is helpful in observing the influence of the parameters on the shape of the solutions. The idea of using small amounts of spatial grid points and saving time for computing solutions of the model equations is crucial in the effective utilization of, for example, animated simulations of tumours, which can be used as a predictive and visualized tool in clinical applications. Decreasing the amounts of spatial grid points used for such visualizations saves not only the time of demonstrations but also the computer memory. It is not possible to demonstrate the animated simulations in papers and we only note that they are interesting and help in visualization of the complicated biological processes. Instead of the animated simulations we include snapshots at different stages in time.

The contents of this paper is as follows: the model equations are described in Section 2, the algorithm is introduced in Section 3, the results of numerical experiments and simulations are presented in Section 4, and Section 5 includes our concluding remarks and future research work.

## 2. Mathematical Model

In this section, we investigate various models of tissue invasion by cancerous cells. In Section 2.1, we investigate the Chaplain and Anderson model [34] focusing on interactions between ECM and cancer tumour and metastatic abilities of cancer cells. In Section 2.2, we investigate further expansions of the model and its different versions with additional terms connected with proliferation of tumour cells, ECM renewal, and different functions modelling the production of MDEs by the tumour cells.  Section 2.3 deals with a more general model with an additional equation, which describes evolution of endogenous inhibitors.

### 2.1. Cell-Matrix Interactions and Cell Migration

In the next section, we construct a numerical scheme for the following model of tissue invasion:

(1) $$\begin{array}{c} \frac{\partial n}{\partial t}=d_{n}\frac{\partial^{2}n}{\partial x^{2}}-\gamma \frac{\partial }{\partial x}\left(n\frac{\partial f}{\partial x}\right),\\ \frac{\partial f}{\partial t}=-\eta mf,\\ \frac{\partial m}{\partial t}=d_{m}\frac{\partial^{2}m}{\partial x^{2}}+\alpha n-\beta m \end{array}$$

with the space variable *x* belonging to the scaled domain [0,1] of tissue, and time *t*. The model equations (1) describe interactions between tumour cells, MDEs, and ECM. The interacting variables are *n*-tumour cell density, *f*-ECM density, and *m*-MDEs concentration. The system (1) is derived in detail in [34] and is a part of a more general system consisting of (1) with an additional fourth equation for endogenous inhibitor concentration denoted by *u*. In [34], it is assumed that the tumour cells, the MDEs, and the inhibitors remain within the space domain and zero-flux boundary conditions are imposed. The fourth equation for the endogenous inhibitor concentration is dropped under the additional assumption that negative effect of the endogenous inhibitors is overcame by the MDEs in an actively invading tumour. This assumption implies that *u* = 0 and the general system of four equations is reduced to (1). In Section 2.3, we investigate the model with all four equations.

### 2.2. Migration and Proliferation of Cancer Cells, ECM Renewal, and MDE Production

We also investigate further expansions of the model (1), which are introduced, for example, in [36, 37]. After adding the proliferation term *μ*_1_ *n*(1 − *n* − *f*) to the right-hand side of the equation governing tumour cell motion (the first equation in (1)) and the ECM renewal term *μ*_2_ *f*(1 − *n* − *f*) to the right-hand side of the equation for the ECM (the second equation in (1)), we obtain the following model:

(2) $$\begin{array}{cc} \frac{\partial n}{\partial t} & =d_{n}\frac{\partial^{2}n}{\partial x^{2}}-\gamma \frac{\partial }{\partial x}\left(n\frac{\partial f}{\partial x}\right)+\mu_{1}n\left(1-n-f\right),\\ \frac{\partial f}{\partial t} & =-\eta mf+\mu_{2}f\left(1-n-f\right),\\ \frac{\partial m}{\partial t} & =d_{m}\frac{\partial^{2}m}{\partial x^{2}}+\alpha n-\beta m, \end{array}$$

where *μ*_1_ is the proliferation rate of the tumour cells and *μ*_2_ is the growth rate of the ECM.

We also make experiments with the following modification of (2):

(3) $$\begin{array}{cc} \frac{\partial n}{\partial t} & =d_{n}\frac{\partial^{2}n}{\partial x^{2}}-\gamma \frac{\partial }{\partial x}\left(n\frac{\partial f}{\partial x}\right)+\mu_{1}n\left(1-n-f\right),\\ \frac{\partial f}{\partial t} & =-\eta mf+\mu_{2}f\left(1-n-f\right),\\ \frac{\partial m}{\partial t} & =d_{m}\frac{\partial^{2}m}{\partial x^{2}}+\alpha n\left(1-n\right)-\beta m, \end{array}$$

where the MDE production is modeled by *αn*(1 − *n*). The motivation for choosing such form of the MDE production in [36, 37] follows from experimental observations of polarized expression of MDEs at the invading leading edge of tumour, see, for example, Estreicher et al. [46].

We investigate the model equations (1), (2), and (3) supplemented by the zero-flux boundary conditions

(4) $$\begin{array}{c} \frac{\partial n}{\partial x}\left(0,t\right)=\frac{\gamma }{d_{n}}n\left(0,t\right)\frac{\partial f}{\partial x}\left(0,t\right),\;\;\frac{\partial m}{\partial x}\left(0,t\right)=0 \end{array}$$

at *x* = 0 and either the Dirichlet conditions

(5) $$\begin{array}{c} n\left(1,t\right)=0,\;\;m\left(1,t\right)=0 \end{array}$$

or the zero-flux boundary condition

(6) $$\begin{array}{c} \frac{\partial n}{\partial x}\left(1,t\right)=\frac{\gamma }{d_{n}}n\left(1,t\right)\frac{\partial f}{\partial x}\left(1,t\right),\;\frac{\partial m}{\partial x}\left(1,t\right)=0, \end{array}$$

at *x* = 1. As in [34], we assume that the initial tumour is centered at *x* = 0, the initial MDE concentration is proportional to the initial tumour cell density with 1/2 as the constant of the proportionality, and the MDE has already degraded the ECM, thus we consider the same initial conditions as in [34], which are the following:

(7) $$\begin{array}{cc} n\left(x,0\right) & =\exp\;\;\left(\frac{-x^{2}}{ɛ}\right),\\ f\left(x,0\right) & =1-0.5n\left(x,0\right),\\ m\left(x,0\right) & =0.5n\left(x,0\right), \end{array}$$

for *x* ∈ [0,1]. The parameter values for the model equations are specified in Section 4.

### 2.3. Production of Endogenous Inhibitors

We additionally consider the general model

(8) $$\begin{array}{cc} \frac{\partial n}{\partial t} & =d_{n}\frac{\partial^{2}n}{\partial x^{2}}-\gamma \frac{\partial }{\partial x}\left(n\frac{\partial f}{\partial x}\right)+\mu_{1}n\left(1-n-f\right),\\ \frac{\partial f}{\partial t} & =-\eta mf+\mu_{2}f\left(1-n-f\right),\\ \frac{\partial m}{\partial t} & =d_{m}\frac{\partial^{2}m}{\partial x^{2}}+\alpha n-\theta um-\beta m,\\ \frac{\partial u}{\partial t} & =d_{u}\frac{\partial^{2}u}{\partial x^{2}}+F\left(m,f\right)-\theta um-\rho u, \end{array}$$

where the last equation describes evolution of endogenous inhibitors (concentration of which is denoted by *u*). This equation is the fourth equation in the model (10.5) proposed by Chaplain and Anderson in [34], where it is assumed that endogenous inhibitors are produced by ECM as a response to the MDEs and the function *F*(*m*, *f*) models the inhibitor production. The term *θum* models neutralization of the MDEs and *ρu* models decay of the inhibitors. We assume that the initial inhibitor concentration is

(9) $$\begin{array}{c} u\left(x,0\right)=0 \end{array}$$

and impose the zero-flux boundary conditions

(10) $$\begin{array}{c} \frac{\partial u}{\partial x}\left(0,t\right)=\frac{\partial u}{\partial x}\left(1,t\right)=0. \end{array}$$

Our goal is to construct a new efficient algorithm for solving the models (1), (2), (3), and (8) and investigate the ability of cancer cells to produce and secrete the MDE, which then degrade the ECM, and allow the cells to start their migration towards healthy parts of the tissue.

## 3. Construction of Numerical Approximations to Tumour Cells, ECM, and MDEs

In this section, we construct numerical solutions to the model equations (1), (2), and (3) supplemented by the initial conditions (7) and the boundary conditions (4) and (5). For the numerical solutions, we consider the Chebyshev-Gauss-Lobatto points

(11) $$\begin{array}{c} x_{i}=\frac{1}{2}-\frac{1}{2}\cos\;\frac{i\pi }{N+1}, \end{array}$$

with *i* = 0,1,…, *N* + 1, in the scaled domain [0,1] of tissue. Our goal is to construct approximations to *n*(*x*_*i*_, *t*), *f*(*x*_*i*_, *t*), and *m*(*x*_*i*_, *t*), for *i* = 0,1,…, *N*; the values of the solutions at *x*_*N*+1_ are known from (5).

Let

(12) $$\begin{array}{c} n\left(t\right)=\left[\begin{array}{c} n\left(x_{0},t\right)\\ n\left(x_{1},t\right)\\ \vdots \\ n\left(x_{N},t\right) \end{array}\right],\;\;\frac{dn\left(t\right)}{dt}=\left[\begin{array}{c} \frac{\partial n}{\partial t}\left(x_{0},t\right)\\ \frac{\partial n}{\partial t}\left(x_{1},t\right)\\ \vdots \\ \frac{\partial n}{\partial t}\left(x_{N},t\right) \end{array}\right],\\ n_{x}\left(t\right)=\left[\begin{array}{c} \frac{\partial n}{\partial x}\left(x_{0},t\right)\\ \frac{\partial n}{\partial x}\left(x_{1},t\right)\\ \vdots \\ \frac{\partial n}{\partial \text{x}}\left(x_{N},t\right) \end{array}\right],\;\;n_{xx}\left(t\right)=\left[\begin{array}{c} \frac{\partial^{2}n}{\partial x^{2}}\left(x_{0},t\right)\\ \frac{\partial^{2}n}{\partial x^{2}}\left(x_{1},t\right)\\ \vdots \\ \frac{\partial^{2}n}{\partial x^{2}}\left(x_{N},t\right) \end{array}\right], \end{array}$$

and we use similar notations for *f* and *m*. We shall replace the spatial derivatives in (1) by numerical approximations constructed for the vectors *n*_*xx*_(*t*), *n*_*x*_(*t*), *f*_*x*_(*t*), *f*_*xx*_(*t*) in the first equation and for *m*_*xx*_(*t*) in the third equation. For *n*_*x*_(*t*), we apply the following spectrally accurate approximations

(13) $$\begin{array}{c} \frac{\partial n}{\partial x}\left(x_{i},t\right)\approx \overset{N+1}{\underset{j=0}{\sum }}d_{i,j}n\left(x_{j},t\right), \end{array}$$

with *i* = 0,1,…, *N* + 1, where

(14) $$\begin{array}{c} D={\left[d_{i,j}\right]}_{i,j=0}^{N+1} \end{array}$$

is the first-order differentiation matrix based on the points (11), see [47, 48]. We also apply the analogous spectrally accurate approximations

(15) $$\begin{array}{c} \frac{\partial f}{\partial x}\left(x_{i},t\right)\approx \overset{N+1}{\underset{j=0}{\sum }}d_{i,j}f\left(x_{j},t\right),\;\;\frac{\partial m}{\partial x}\left(x_{i},t\right)\approx \overset{N+1}{\underset{j=0}{\sum }}d_{i,j}m\left(x_{j},t\right) \end{array}$$

for *f*_*x*_(*t*) and *m*_*x*_(*t*), respectively.

Since the exact value of (∂*n*/∂*x*)(*x*_0_, *t*) is given by (4), the approximation (13) is not needed at the first point *x*_0_, that is, for the first component of *n*_*x*_(*t*). Therefore, from (13), the first approximation in (15) with *f* and *i* = 0, and from the boundary conditions (4) and (5) we obtain

(16) $$\begin{array}{c} n_{x}\left(t\right)\approx D_{0}^{\left(1\right)}n\left(t\right)+\frac{\gamma }{d_{n}}n\left(x_{0},t\right)s_{0}^{f}\left(t\right)e_{1}, \end{array}$$

where

(17) $$\begin{array}{c} D_{0}^{\left(1\right)}=\left[\begin{array}{cccc} 0 & 0 & \cdots & 0\\ d_{1,0} & d_{1,1} & \cdots & d_{1,N}\\ \vdots & \vdots & \ddots & \vdots \\ d_{N,0} & d_{N,1} & \cdots & d_{N,N} \end{array}\right],\;\;e_{1}=\left[\begin{array}{c} 1\\ 0\\ \vdots \\ 0 \end{array}\right],\\ s_{0}^{f}\left(t\right)=\overset{N+1}{\underset{j=0}{\sum }}d_{0,j}f\left(x_{j},t\right). \end{array}$$

From (13) and (16) we obtain the following approximation for the second-order derivatives

(18) $$\begin{array}{cc} n_{xx}\left(t\right) & \approx D^{\left(1\right)}n_{x}\left(t\right)+s_{N+1}^{n}\left(t\right)w\\ & \approx D^{\left(1\right)}\left(D_{0}^{\left(1\right)}n\left(t\right)+\frac{\gamma }{d_{n}}n\left(x_{0},t\right)s_{0}^{f}\left(t\right)e_{1}\right)+s_{N+1}^{n}\left(t\right)w, \end{array}$$

where

(19) $$\begin{array}{c} D^{\left(1\right)}=\left[\begin{array}{cccc} d_{0,0} & d_{0,1} & \cdots & d_{0,N}\\ d_{1,0} & d_{1,1} & \cdots & d_{1,N}\\ \vdots & \vdots & \ddots & \vdots \\ d_{N,0} & d_{N,1} & \cdots & d_{N,N} \end{array}\right],\;\;w=\left[\begin{array}{c} d_{0,N+1}\\ d_{1,N+1}\\ \vdots \\ d_{N,N+1} \end{array}\right],\\ s_{N+1}^{n}\left(t\right)=\overset{N+1}{\underset{j=0}{\sum }}d_{N+1,j}n\left(x_{j},t\right). \end{array}$$

We now construct approximations to *f*_*x*_(*t*), *m*_*x*_(*t*), *f*_*xx*_(*t*), and *m*_*xx*_(*t*). From the spectrally accurate approximations (15) and from (4) and (5) we obtain

(20) $$\begin{array}{c} f_{x}\left(t\right)\approx D^{\left(1\right)}f\left(t\right)+f\left(x_{N+1},t\right)w, \end{array}$$

(21) $$\begin{array}{c} m_{x}\left(t\right)\approx D_{0}^{\left(1\right)}m\left(t\right). \end{array}$$

According to (20), we have the following approximation for the second-order derivative of *f*

(22) $$\begin{array}{c} f_{xx}\left(t\right)\approx D^{\left(1\right)}\left(D^{\left(1\right)}f\left(t\right)+f\left(x_{N+1},t\right)w\right)+s_{N+1}^{f}\left(t\right)w, \end{array}$$

with the notation

(23) $$\begin{array}{c} s_{N+1}^{f}\left(t\right)=\overset{N+1}{\underset{j=0}{\sum }}d_{N+1,j}f\left(x_{j},t\right). \end{array}$$

From (21) we have

(24) $$\begin{array}{c} m_{xx}\left(t\right)\approx D^{\left(1\right)}D_{0}^{\left(1\right)}m\left(t\right)+s_{N+1}^{m}\left(t\right)w, \end{array}$$

with the similar notation for *m*

(25) $$\begin{array}{c} s_{N+1}^{m}\left(t\right)=\overset{N+1}{\underset{j=0}{\sum }}d_{N+1,j}m\left(x_{j},t\right). \end{array}$$

We now replace the spatial derivatives in the model (1) by their corresponding approximations. We apply (18), (16), (20), and (22) to the first equation in (1) and obtain its discrete version written in the following form

(26) $$\begin{array}{cc} \frac{dn}{dt}\left(t\right) & =d_{n}D^{\left(1\right)}\left(D_{0}^{\left(1\right)}n\left(t\right)+\frac{\gamma }{d_{n}}n\left(x_{0},t\right)s_{0}^{f}\left(t\right)e_{1}\right)+d_{n}s_{N+1}^{n}\left(t\right)w\\ & \;-\gamma [\left(D_{0}^{\left(1\right)}n\left(t\right)+\frac{\gamma }{d_{n}}n\left(x_{0},t\right)s_{0}^{f}\left(t\right)e_{1}\right)\\ & \;\;\;⊙\left(D^{\left(1\right)}f\left(t\right)+f\left(x_{N+1},t\right)w\right)+n\left(t\right)\\ & \;\;\;⊙\left(D^{\left(1\right)}\left(D^{\left(1\right)}f\left(t\right)+f\left(x_{N+1},t\right)w\right)+s_{N+1}^{f}\left(t\right)w\right)], \end{array}$$

where ⊙ stands for the component-wise multiplication between two vectors. The discrete version of the second equation in (1) is written in the form

(27) $$\begin{array}{c} \frac{df}{dt}\left(t\right)=-\eta \left(m\left(t\right)⊙f\left(t\right)\right) \end{array}$$

and from (24) we obtain the following discrete form of the third equation in  (1)

(28) $$\begin{array}{c} \frac{dm}{dt}\left(t\right)=d_{m}D^{\left(1\right)}D_{0}^{\left(1\right)}m\left(t\right)+d_{m}s_{N+1}^{m}\left(t\right)w+\alpha n\left(t\right)-\beta m\left(t\right). \end{array}$$

The resulting system (26)–(28) is composed of 3*N* + 3 ordinary differential equations and is a semidiscrete version of (1). Note that since the spatial derivatives in (1) are approximated with the spectral accuracy, much smaller numbers of grid-points *x*_*i*_ are needed for (26)–(28) than for finite difference schemes, and time integration of the smaller systems is more robust and more efficient than time integration of the finite difference systems.

For the models (2) and (3) supplemented with (4) and the right-hand side boundary condition (6), which is different than (5), we need to apply different approximations than (16), (18), (20), and (22) as they include (5) instead of (6). For this problem, from (13), instead of (16), we obtain

(29) $$\begin{array}{c} n_{x}\left(t\right)\approx D_{00}^{\left(1\right)}n\left(t\right)+\frac{\gamma }{d_{n}}w^{\left(0,N+1\right)}, \end{array}$$

where

(30) $$\begin{array}{c} D_{00}^{\left(1\right)}=\left[\begin{array}{cccc} 0 & 0 & \cdots & 0\\ d_{1,0} & d_{1,1} & \cdots & d_{1,N}\\ \vdots & \vdots & \ddots & \vdots \\ d_{N,0} & d_{N,1} & \cdots & {\text{d}}_{N,N}\\ 0 & 0 & \cdots & 0 \end{array}\right],\\ w^{\left(0,N+1\right)}=\left[\begin{array}{c} s_{0}^{f}\left(t\right)n\left(x_{0},t\right)\\ 0\\ \vdots \\ 0\\ s_{N+1}^{f}\left(t\right)n\left(x_{N+1},t\right) \end{array}\right]. \end{array}$$

Further, from (29), we obtain

(31) $$\begin{array}{c} n_{xx}\left(t\right)\approx D^{\left(1\right)}\left(D_{00}^{\left(1\right)}n\left(t\right)+\frac{\gamma }{d_{n}}w^{\left(0,N+1\right)}\right). \end{array}$$

As in (13) and (15), for the vector

(32) $$\begin{array}{c} ℋ\left(t\right)=\left[\begin{array}{c} \frac{\partial }{\partial x}\left(n\left(x_{0},t\right)\frac{\partial f}{\partial x}\left(x_{0},t\right)\right)\\ \frac{\partial }{\partial x}\left(n\left(x_{1},t\right)\frac{\partial f}{\partial x}\left(x_{1},t\right)\right)\\ \vdots \\ \frac{\partial }{\partial x}\left(n\left(x_{N},t\right)\frac{\partial f}{\partial x}\left(x_{N},t\right)\right) \end{array}\right], \end{array}$$

of approximations to the haptotactic term we obtain

(33) $$\begin{array}{c} ℋ\left(t\right)\approx D^{\left(1\right)}\left(n\left(t\right)⊙f_{x}\left(t\right)\right) \end{array}$$

and instead of (24), from (6), we obtain

(34) $$\begin{array}{c} m_{xx}\left(t\right)\approx D_{00}^{\left(1\right)}m\left(t\right). \end{array}$$

From (29), (31), (33), and (34) we obtain the following scheme for the problem (3), (4), and  (6)

(35) $$\begin{array}{cc} \frac{dn}{dt}\left(t\right) & =D^{\left(1\right)}\left(d_{n}D_{00}^{\left(1\right)}n\left(t\right)+\gamma w^{\left(0,N+1\right)}-\gamma n\left(t\right)⊙f_{x}\left(t\right)\right)\\ & \;+\mu_{1}n\left(t\right)⊙\left(e-n\left(t\right)-f\left(t\right)\right),\\ \frac{df}{dt}\left(t\right) & =-\eta \left(m\left(t\right)⊙f\left(t\right)\right)+\mu_{2}f\left(t\right)⊙\left(e-n\left(t\right)-f\left(t\right)\right),\\ \frac{dm}{dt}\left(t\right) & =d_{m}D_{00}^{\left(1\right)}m\left(t\right)+\alpha n\left(t\right)⊙\left(e-n\left(t\right)\right)-\beta m\left(t\right), \end{array}$$

where *e* is a vector entries of which are all 1-s. For (2), the component *αn*(*t*) ⊙ (*e* − *n*(*t*)) needs to be replaced by *αn*(*t*) in the last equation of (35). From the boundary conditions (10), we obtain the approximation for the diffusion of the endogenous inhibitors

(36) $$\begin{array}{c} u_{xx}\left(t\right)\approx D_{00}^{\left(1\right)}u\left(t\right) \end{array}$$

and the semi-discrete version for the last equation in (8) is written in the following form

(37) $$\begin{array}{c} \frac{du}{dt}\left(t\right)=d_{u}D_{00}^{\left(1\right)}u\left(t\right)+\xi f\left(t\right)-\theta u\left(t\right)⊙m\left(t\right)-\rho u\left(t\right), \end{array}$$

where we assume that the inhibitor production is modelled by *F*(*m*, *f*) = *ξf*. The semi-discrete equations have to be closed by initial conditions chosen according to (7) and (9).

## 4. Numerical Experiments

**Figure 1:**
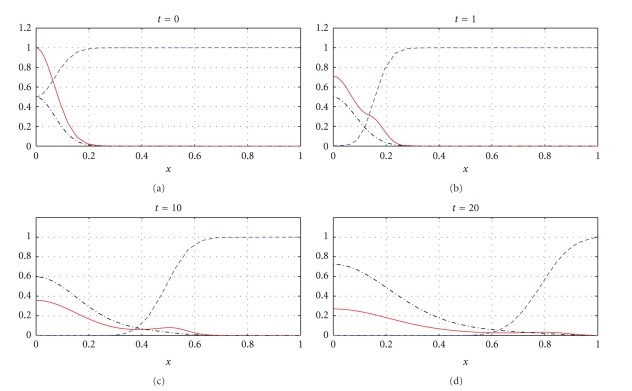
Tumour cell migration and interactions between the tumour and the surrounding tissue: tumour cell density (solid), ECM density (dashed), and MDE concentration (dashdot). Solutions to (1), (4), (5), (7) with the parameter values *γ* = 0.005 and *η* = 10.

**Figure 2:**
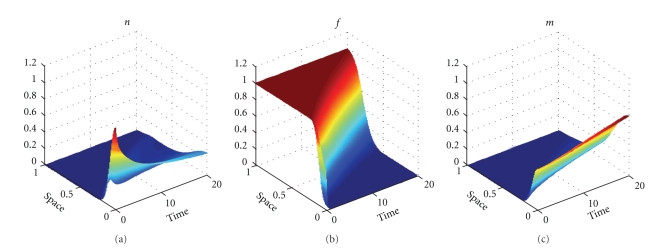
Evolution in time: the tumour cell density (a), ECM density (b), and MDE concentration (c). Solutions to (1), (4), (5), (7) with the parameter values as in Figure 1.

**Figure 3:**
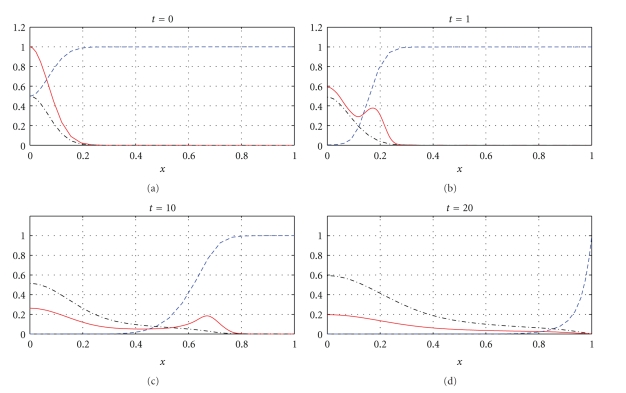
Tumour cell migration and interactions between the tumour and the surrounding tissue: tumour cell density (solid), ECM density (dashed), and MDE concentration (dashdot). Solutions to (1), (4), (5), (7) with the parameter values *γ* = 0.01 and *η* = 10.

**Figure 4:**
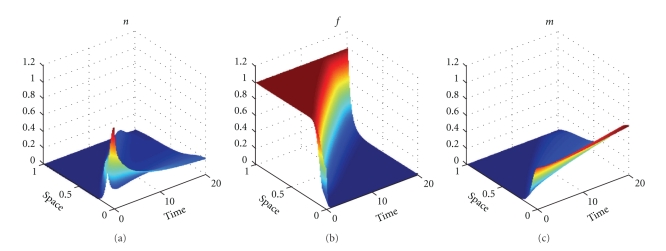
Evolution in time: the tumour cell density (a), ECM density (b), and MDE concentration (c). Solutions to (1), (4), (5), (7) with the parameter values as in Figure 3.

**Figure 5:**
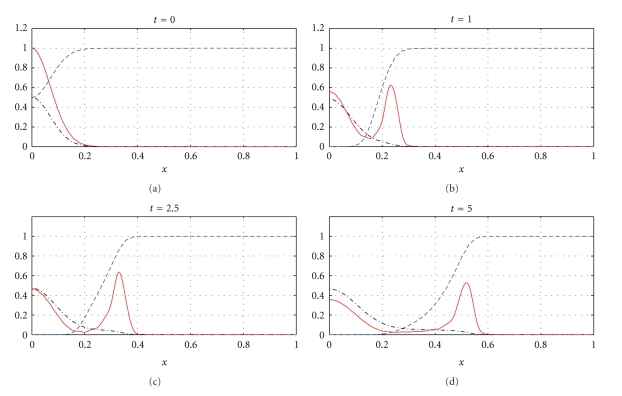
Tumour cell migration and interactions between the tumour and the surrounding tissue: tumour cell density (solid), ECM density (dashed), and MDE concentration (dashdot). Solutions to (1), (4), (5), (7) with the parameter values *γ* = 0.02 and *η* = 20.

**Figure 6:**
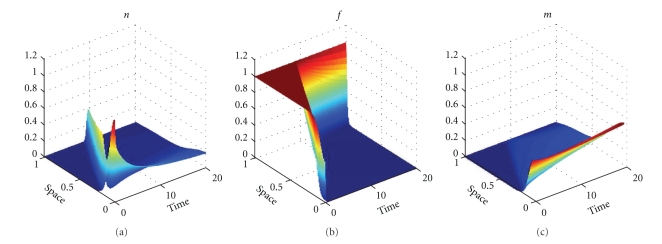
Evolution in time: the tumour cell density (a), ECM density (b), and MDE concentration (c). Solutions to (1), (4), (5), (7) with the parameter values as in Figure 5.

We apply the approximations introduced in Section 3 and begin our series of numerical simulations from (26)–(28), which correspond to model (1). Results of our numerical experiments are presented in Figures 1–6. We use the parameter values *d*_*n*_ = 0.001, *d*_*m*_ = 0.001, *α* = 0.1, *β* = 0, *ɛ* = 0.01, and different values of *γ* and *η* specified in the captions of the figures.

We apply *N* = 30, that is 32 grid-points *x*_*i*_, for the numerical experiments presented in Figures 1–4. The time of integration of the system (26)–(28) based on 32 grid points is 0.22 sec to compute the numerical solutions presented in Figures 1 and 2 and 0.38 sec to compute the numerical solutions from Figures 3 and 4. For Figures 5 and 6, we apply *N* = 43, that is 45 grid points, and in this case, the time of integration of (26)–(28) is 0.35 sec.

Figures 1, 3, and 5 show snapshots in time and Figures 2, 4, and 6 show continuous evolution in time of tumour cells, ECM, and MDE, and their interactions for all *x* in the space domain. The numerical results presented in Figures 1 and 2 were obtained with *γ* = 0.005 and *η* = 10, see also [34, Figure 10.2]. The results from Figures 3 and 4 were obtained with *γ* = 0.01 and *η* = 10.

Two distinct clusters of tumour cells are seen in Figures 1 and 3 at *t* = 1 and *t* = 10. The numerical results show that the new clusters, which are not seen at *t* = 0 and appear at *t* = 1 and *t* = 10, are created at the leading edge of the tumour as a result of the diffusion and haptotactic migration modeled by the two components from the right-hand side of the first equation in (1): random motility *d*_*n*_Δ^2^ *n* and haptotaxis −*γ*Δ · (*n*Δ*f*), respectively. Since *γ* is greater for Figure 3 than for Figure 1, because of larger haptotactic migration in Figure 3 than in Figure 1, the two clusters seen in Figure 3 are more separated from each other than the two clusters in Figure 1. The pictures show the effect of haptotaxis. The small clusters of cells, which break away from the main body of the tumour, illustrate the potential for the cancer cells to degrade the surrounding tissue, migrate, and start the metastatic cascade. The migrations of the small clusters may not be detected during the processes of medical treatments, and even after resections of the main tumours, the new small clusters may initiate recurrences of the disease. A new cluster of tumour cells broken away from the main body of the tumour is also observed in Figures 5 and 6, which present numerical data computed with *γ* = 0.02 and *η* = 20.

**Figure 7:**
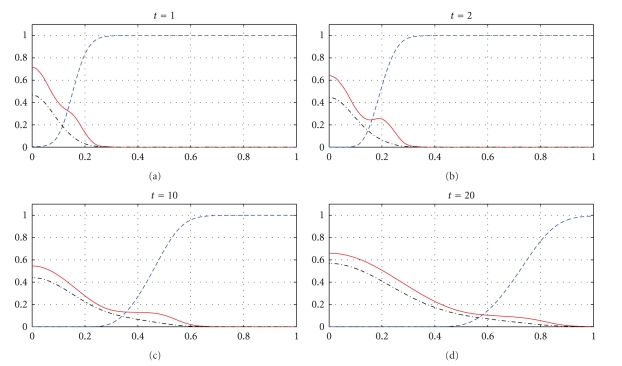
Tumour cell proliferation, migration, ECM re-establishment, and interactions between the tumour and the surrounding tissue: tumour cell density (solid), ECM density (dashed), and MDE concentration (dashdot). Solutions to (2), (4), (6), (7) with the parameter values *γ* = 0.005, *η* = 10, *μ*_1_ = 0.1, *μ*_2_ = 0.5, and *β* = 0.07.

**Figure 8:**
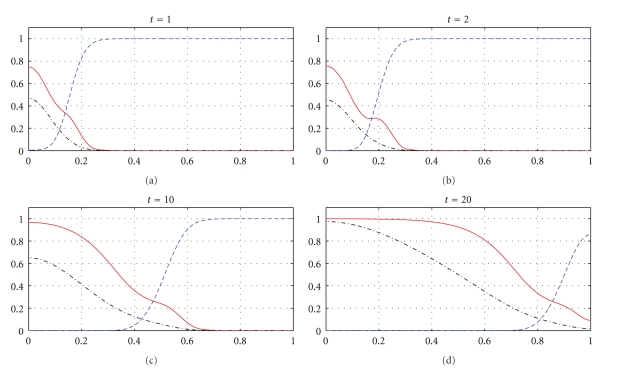
Tumour cell proliferation, migration, ECM re-establishment, and interactions between the tumour and the surrounding tissue: tumour cell density (solid), ECM density (dashed), and MDE concentration (dashdot). Solutions to (2), (4), (6), (7) with the parameter values *γ* = 0.005, *η* = 10, *μ*_1_ = 0.5, *μ*_2_ = 0.1, and *β* = 0.07.

**Figure 9:**
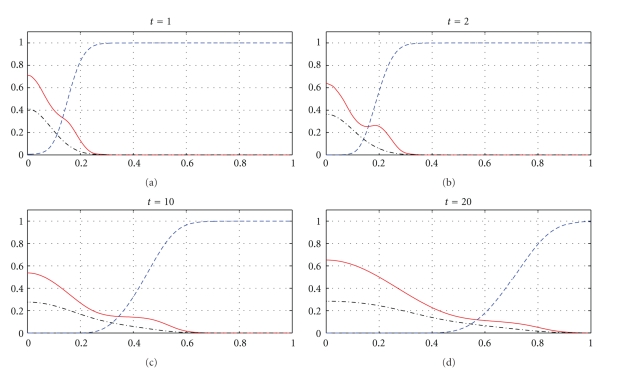
Tumour cell proliferation, migration, ECM re-establishment, and interactions between the tumour and the surrounding tissue: tumour cell density (solid), ECM density (dashed), and MDE concentration (dashdot). Solutions to (3), (4), (6), (7) with the parameter values *γ* = 0.005, *η* = 10, *μ*_1_ = 0.1, *μ*_2_ = 0.5, and *β* = 0.07.

**Figure 10:**
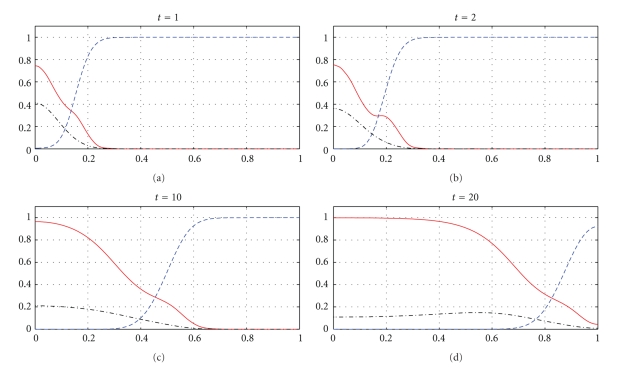
Tumour cell proliferation, migration, ECM re-establishment, and interactions between the tumour and the surrounding tissue: tumour cell density (solid), ECM density (dashed), and MDE concentration (dashdot). Solutions to (3), (4), (6), (7) with the parameter values *γ* = 0.005, *η* = 10, *μ*_1_ = 0.5, *μ*_2_ = 0.1, and *β* = 0.07.

**Figure 11:**
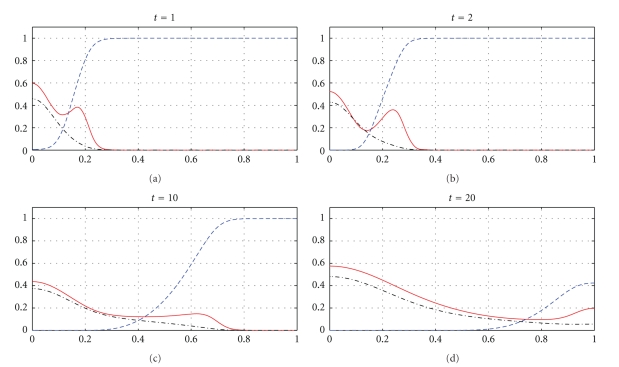
Tumour cell proliferation, migration, ECM re-establishment, and interactions between the tumour and the surrounding tissue: tumour cell density (solid), ECM density (dashed), and MDE concentration (dashdot). Solutions to (2), (4), (6), (7) with the parameter values *γ* = 0.01, *η* = 10, *μ*_1_ = 0.1, *μ*_2_ = 0.5, and *β* = 0.07.

**Figure 12:**
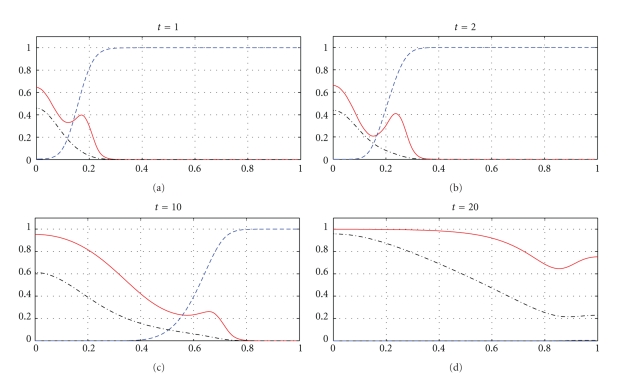
Tumour cell proliferation, migration, ECM re-establishment, and interactions between the tumour and the surrounding tissue: tumour cell density (solid), ECM density (dashed), and MDE concentration (dashdot). Solutions to (2), (4), (6), (7) with the parameter values *γ* = 0.01, *η* = 10, *μ*_1_ = 0.5, *μ*_2_ = 0.1, and *β* = 0.07.

**Figure 13:**
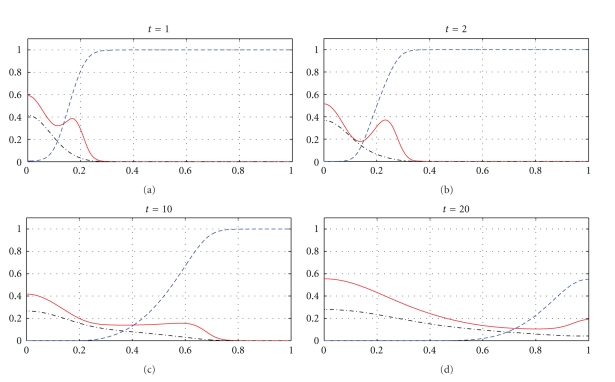
Tumour cell proliferation, migration, ECM re-establishment, and interactions between the tumour and the surrounding tissue: tumour cell density (solid), ECM density (dashed), and MDE concentration (dashdot). Solutions to (3), (4), (6), (7) with the parameter values *γ* = 0.01, *η* = 10, *μ*_1_ = 0.1, *μ*_2_ = 0.5, and *β* = 0.07.

**Figure 14:**
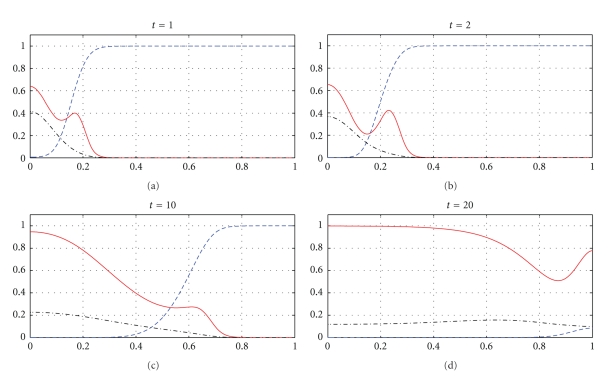
Tumour cell proliferation, migration, ECM re-establishment, and interactions between the tumour and the surrounding tissue: tumour cell density (solid), ECM density (dashed), and MDE concentration (dashdot). Solutions to (3), (4), (6), (7) with the parameter values *γ* = 0.01, *η* = 10, *μ*_1_ = 0.5, *μ*_2_ = 0.1, and *β* = 0.07.

The next part of our numerical experiments concerns the models (2) and (3) supplemented by (4), (6), and (7). The results for model (2) are presented in Figures 7, 8, 11, and 12 and for model (3) in Figures 9, 10, 13, and 14. These experiments start from the initial condition (7) corresponding to the snapshot in time *t* = 0 in Figure 1. We observe that the small clusters of cancer cells separated from the main tumours are better formed at *t* = 2 than at *t* = 1 and as time evolves the haptotactic migration together with the production of new cancer cells spread the shapes of the tumours over the *x*-domain. We also observe that the snapshots in time *t* = 1 in Figures 1, 7, and 8 look similar to each other and the models (1), (2), and (3) give similar results for *t* ∈ [0,1] although they are supplemented by the different boundary conditions, either (5) or (6), and solved with different parameters *μ*_1_, *μ*_2_ ∈ {0,0.1,0.5} and *β* ∈ {0,0.07}. However, these similarities are observed only for *t* ∈ [0,1] and as time evolves the corresponding solutions of the models (1), (2), and (3) differ from each other. For example, already at *t* = 2, Figure 8 shows greater production of tumour cells than Figure 7. Moreover, at *t* = 10 and *t* = 20, Figure 7 shows weaker MDE production and greater production of the tumour cells and the ECM than in Figure 1 due to the fact that *μ*_1_, *μ*_2_, and *β* are greater in Figure 7 than in Figure 1. On the other hand, also at *t* = 10 and *t* = 20, Figure 8 shows greater MDE production than Figure 1. Although *β* = 0.07 for Figure 8 and *β* = 0 for Figure 1, since the MDE production is greater in Figure 8 than in Figure 1, the MDE concentration is greater in Figure 8 than in Figure 1 and consequently the ECM degradation is more progressive in Figure 8 than in Figure 1. It can also be observed that although the parameters *d*_*m*_, *α*, and *β* in the third equation of (1) and (2) are the same for Figures 1, 7, and 8, the MDE curves show different MDE concentrations in all of these figures.

In Figures 7–10, we observe differences due to the MDE production terms *αn* and *αn*(1 − *n*) in (2) and (3), respectively. The parameter values for Figure 9 are the same as for Figure 7 and the parameter values for Figure 10 are the same as for Figure 8 but the MDE production is weaker in Figure 9 than in Figure 7 and also weaker in Figure 10 than in Figure 8. The MDE concentrations in Figures 9 and 10 are more uniformly spread out across the *x*-domain than in Figures 7 and 8. Furthermore, Figures 9 and 10 illustrate that the system (3) models a decreasing MDE production as the tumour invasion progresses and consequently less MDE concentrations in the regions where the high tumour cells densities are situated (possibly in these regions the tumour cells already finalized their invasion due to lack of space and move to other regions) than in the regions of less advanced stage of invasion. This shows that cancer cells may not need MDEs in the regions where they deal with lack of space. Similar features are observed in Figures 11–14, where the clusters separating from the main tumours are more profound than in Figures 7–10. The same parameter values were used for Figure 11 as for Figure 13 and the same parameter values were used for Figure 12 as for Figure 14. Figures 11 and 12 illustrate numerical solutions to (2), while Figures 13 and 14 illustrate numerical solutions to (3). Comparison of Figures 11–14 confirms that the term *αn*(1 − *n*) in (3) models lower MDE production than the term *αn* in (2) (with the same parameter values). We also observe that since MDE production is lower in Figures 13 and 14 than in Figures 11 and 12, the ECM degradation is smaller in Figures 13 and 14 than in Figures 11 and 12.

**Figure 15:**
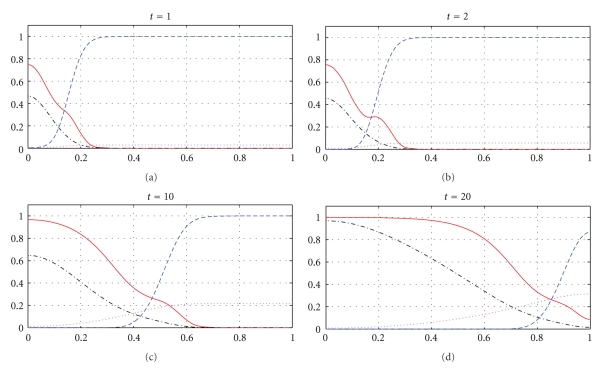
Tumour cell density (solid), ECM density (dashed), MDE concentration (dashdot), and endogenous inhibitors (dotted). Solutions to (8) with (4), (6), (7), (9), (10) and the parameter values *γ* = 0.005, *η* = 10, *μ*_1_ = 0.5, *μ*_2_ = 0.1, *β* = 0.07, *d*_*u*_ = *d*_*n*_ = *d*_*m*_ = 0.001, *θ* = 0.05, *ξ* = 0.03, and *ρ* = 0.07.

Figure 15 shows snapshots in time of the four solutions to the more general model (8) describing the interactions between the tumour cells, ECM, MDEs, and endogenous inhibitors. All four profiles show that, as time evolves, the ECM produces endogenous inhibitors, concentration of which increases in time and their higher concentration is located in the regions where ECM is not yet entirely degraded rather than in the regions where the degradation is already effectively developed. The inhibitor profile shows that the ECM responds to the MDEs by producing the endogenous inhibitors.

## 5. Concluding Remarks and Future Directions

We have constructed a new numerical algorithm for fast computations of the solutions of the mathematical models proposed by Chaplain et al. in [34, 36, 37], which consist of systems of nonlinear partial differential equations describing interactions between tumour cells, ECM, and MDEs. The algorithm is based on spectrally accurate approximations and small amounts of grid points, which results in ordinary differential systems of small dimensions and fast computations. We have applied the algorithm and presented and compared the numerical simulations with a variety of model equations. The simulations demonstrate that the models describe important features of the interactions between tumour cells and the surrounding tissue, and in particular the initiation of a new colony of cells and metastasis.

Our future research work will address the question for which parameter values and domains the model [34] and the kinetic type model proposed in [49] are equivalent. We will also address numerical methods with spectrally accurate approximations for the models with two-dimensional spatial domain and with different kinds of the function *F*(*m*, *f*) modelling the inhibitor production [34].
